# The Craig Colony for Epileptics

**Published:** 1895-01

**Authors:** William P. Spratling

**Affiliations:** Medical Superintendent; Mount Morris, N. Y.


### THE CRAIG COLONY FOR EPILEPTICS.

We have received from our former New York correspondent, Dr. W. P. Spratling, a circular letter descriptive of this institution, of which he is now medical superintendent. The following will be of interest:

MOUNT MORRIS, N. Y., December, 1894.

The Colony consists of 1,856 acres of land, near Mt. Morris, in Livingston county, New York. There are upon it thirty-five or forty buildings, which are being put in order for the accommodation of patients. The Colony was named for Oscar Craig, late President of the State Board of Charities.

The law establishing Craig Colony was passed in the spring of 1894.

The object of the Colony is to provide for the four great needs of epileptics which are not satisfied elsewhere:

1st. To give them schools where they may attain any degree in education.

2d. To provide industrial training of all kinds, for there is no vocation which some epileptics may not follow.

3d. To give them a home when all other doors are closed to them.

4th. To see that each and every case is carefully studied and treated by the best scientific methods the world affords.

Such objects can only be attained in a community, village, or colony devoted to this particular class of cases. There are several such colonies in Europe, but none in this country.

There are about 600 epileptics in the county alms-houses of New York State. There are 400 in the State insane asylum. The Colony is intended to provide for most of these. When the Colony opens, patients from the alms-houses will be the first to be received and these gradually. The law will not permit of any private patients being admitted until all the patients upon public charge are provided for in the Colony.

It is hoped to open the Colony for the reception of a hundred or more patients in the summer of 1895. It is probable that the Colony will ultimately number from 1,500 to 2,000 members, and it is certain to become, in the course of time, a self-supporting industrial and agricultural village. It will more than rival the similar and celebrated Colony at Bielefeld, Germany, upon which it is, to a certain extent, modeled.

The Craig Colony will not resemble an institution in any particular, but will look more like a country town than anything else. As the patients are received they will be set to work or at study in various ways. They will take care of the farms, gardens, and orchards; they will plan and build new houses. There will be among them tailors, shoemakers, printers, book-binders, masons, iron-workers, carpenters, painters, and so on. In fact, every sort of employment, every sort of recreation, everything, in short, that goes to make up the life of any country village, will be found in this Colony, the only difference being, that the citizens of this community will be epileptics.

The resources of the land acquired are such that there is no doubt whatever that, in the course of a few years, this Colony will be more than self-supporting, so that, from the economical standpoint, if not from the philanthropic, the scheme will be a wise one. There are 1,000 epileptics in this State now, in alms-houses and in the asylum, who are a burden to the tax-payers, and these will be taken to the Colony and be made in due time self-supporting.

WILLIAM P. SPRATLING, M.D.,

Medical Superintendent.

